# Death Pathways of Cancer Cells Modulated by Surface Molecule Density on Gold Nanorods

**DOI:** 10.1002/advs.202102666

**Published:** 2021-09-15

**Authors:** Fulei Zhang, Yi Hou, Minhui Zhu, Bo Deng, Mengxin Zhao, Xiandi Zhu, Yun Sun, Di Chen, Cheng Jiang, Liming Wang, Chunying Chen, Huaiwen Chen, Han Chen, Hongliang Zheng, Wei Li

**Affiliations:** ^1^ Department of Nanomedicine & International Joint Cancer Institute Naval Medical University Shanghai 200433 China; ^2^ Department of Otolaryngology Head & Neck Surgery Shanghai Changhai Hospital Naval Medical University 168 Changhai Road Shanghai 200433 China; ^3^ State Key Laboratory of New Textile Materials and Advanced Processing Technologies Wuhan 430073 China; ^4^ CAS Key Laboratory for Biomedical Effects of Nanomaterials and Nanosafety Institute of High Energy Physics and National Center for Nanoscience and Technology of China Chinese Academy of Sciences Beijing 100049 China; ^5^ Department of General Surgery Navy No.905 Hospital of Chinese People's Liberation Army Naval Medical University Shanghai 200050 China

**Keywords:** cancer therapy, cell death pathway, gold nanorods, surface molecule density

## Abstract

Necrosis induces strong inflammation with undesirable implications in clinics compared with apoptosis. Fortunately, the switch between necrosis and apoptosis could be realized by tailoring the appropriate structural properties of gold nano rods (GNRs) that could precisely modulate cell death pathways. Herein, the intracellular interaction between GNRs and organelles is monitored and it is found that lysosomes dominates necrosis/apoptosis evoking. Then the surface molecule density of GNRs, which is first defined as *ρ*_surf. molecule_ (*N*_surf. molecules_/(*a* × *π* × Diameter × Length)), mediates lysosome activities as the membrane permeabilization (LMP), the Cathepsin B and D release, the cross‐talk between lysosome and different organelles, which selectively evokes apoptosis or necrosis and the production of TNF‐*α* from macrophages. GNRs with small *ρ*_surf. molecule_ mainly induce apoptosis, while with large *ρ*_surf. molecule_ they greatly contribute to necrosis. Interestingly, necrosis can be suppressed by GNRs with higher *ρ*_surf. molecule_ due to the overexpression of key protease caspase 8, which cleaves the RIP1‐RIP3 complex and activates caspase 3 followed by necrosis to apoptosis transition. This investigation indicates that the *ρ*_surf. molecule_ greatly affects the utility of nanomaterials and different structural properties of nanomaterials have different implications in clinics.

## Graphical abstract

The cross‐talk between GNRs and subcellular organelles which specifically dominates cancer cell death is detailly discussed in the work. The surface molecule density ****ρ****_surf. molecule_ of GNRs is first defined and derived as ***N***_surf. molecules_/****π*DL*** for mediating lysosome activities. The effects of key Caspase 8 protease related to the necrosis‐to‐apoptosis transition through the ****ρ****_surf. molecule_ is illustrated and discussed.

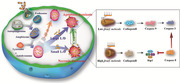

## 1 Introduction

Gold nanorods (GNRs) have been widely used in biomedical fields due to their unique physiochemical properties and biocompatibility.^[^ ^1^, ^2^, ^3^, ^4^, ^5^, ^6^, ^7^, ^8^ ^]^ Combining with positively charged cetyltrimethylammonium bromide (CTAB),^[^ ^9^, ^10^, ^11^, ^12^ ^]^ GNRs kill cancer cells particularly and exert negligible effects on noncancerous cells and stem cells.^[^ ^13^, ^14^, ^15^ ^]^ The specific toxicity of GNRs against cancer cells could be attributed to the strong interaction between the surface molecules of GNRs and the organelles, such as endosome, autophagosome and lysosome.^[^ ^13^, ^16^ ^]^ Thus, when exploring GNRs’ application in cancer therapy, we should first try to focus on their endocytosis, intracellular trafficking, and interactions with suborganelles in cancer cells.

Cell death pathway is pivotal for cancer therapy. In tumor microenvironment, the phagocytosis of the debris produced by necrotic cells stimulates macrophages to release a large amount of TNF‐*α*, which in turn recruit other inflammatory cells from the immune system.^[^ ^17^ ^]^ However, these inflammatory cells could go awry and stimulate tumor development by promoting angiogenesis, proliferation and invasion of cancer cells.^[^ ^18^, ^19^ ^]^ Thus, it is crucial to avoid tumor necrosis during cancer therapy. Because the physical chemical properties of GNRs, such as the aspect ratio and surface composition, determines the interaction between GNRs and lysosomes,^[^ ^1^, ^9^, ^11^, ^15^, ^16^ ^]^ and the interaction dictates cell death pathways, it is critical to design GNRs with proper structural properties, and study their interaction with cellular lysosomes, which could help elucidate the mechanism in which GNRs functions to kill cancer cells and illuminate on how GNRs could be tailored in terms of their physical chemical properties to suppress necrosis and benefit patients more in clinics.

Lysosome and mitochondria are the target organelles of CTAB‐capped GNRs; they are also organelles that are associated with apoptosis and necrosis due to the release of various harmful molecules from lysosomes.^[^ ^9^, ^15^, ^16^ ^]^ Previous studies have found that CTAB‐capped GNRs induce apoptosis. Cancer cell death evoked by GNRs has been attributed to the long‐term intracellular retention of GNRs, which decreases mitochondrial membrane potential and increases reactive oxygen species.^[^ ^7^, ^9^ ^]^ Cathepsin D facilitates mitochondrial outer membrane permeabilization (MOMP) and strongly affects cell apoptosis; whereas Cathepsin B mainly induces necrosis.^[^ ^9^, ^11^, ^15^ ^]^ the cytotoxicity may be due to the block of GNRs in autophagic flux resulting in the degradation of autophagic substrate.^[^ ^14^ ^]^ However, neither the molecular mechanism for cell death nor the detailed regulation of surface chemistry (such as the ligand density) and aspect ratio on cell death pathway is revealed.

To understand the influence that the structural properties of nanomaterials have on cell death pathways is necessary for the design of nanomedicines that could be used in cancer therapy. Herein, we designed two different GNRs (GNR660 and GNR820) with their aspect ratios being GNRs‐660 and GNRs‐820, separately. The CTAB ligand densities on the surfaces of these GNRs differs from each other and the two types of GNRs induces different cell death pathways in cancer cells. Following the characterization of the surface molecule density and surface charges, we studied the influence that these surface properties had on GNRs internalization and localization in human breast adenocarcinoma cells (MDA‐MB‐231), and analyzed the different cell death pathways triggered by different GNRs. We further explored the molecular mechanism how these two GNRs induced differently necrosis and apoptosis of cancer cells. On the basis of the characterization on the surface properties of GNRs with different aspect ratio, we revealed the relationship between physical chemistry properties of GNRs and the necrosis or apoptosis fate of cancer cells.

## 2 Results and Discussion

### 2.1 In Vitro/Vivo Specific Cytotoxicity of GNRs Evaluated by Cancer Cells and Tumor Model

**Figure 1:**
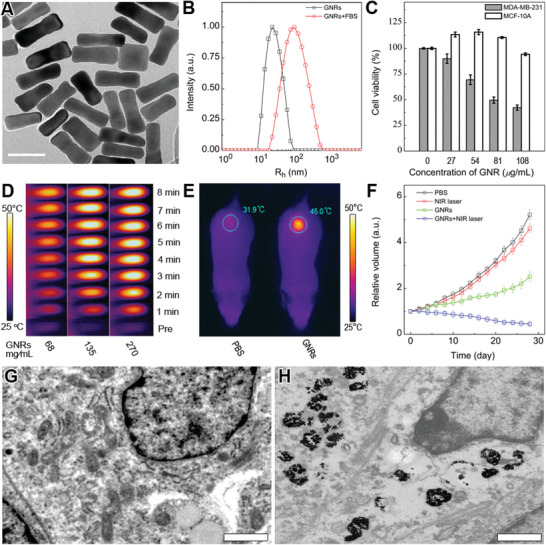
Evaluation of the in vitro*/*vivo cellular cytotoxicity and tumor inhibition activity of GNRs. A) Representative TEM image of GNRs. Scale bar is 50 nm. B) Size distribution of GNRs and GNRs‐FBS by DLS. C) The specificity of CTAB‐GNRs killing cancer cells. D) Characterization of GNRs’ photothermal transition ability. E) Representative images of temperature change in tumor‐bearing mice irradiated by infrared laser. F) Average tumor growth kinetics in different groups. G,H) Representative TEM images of the distribution of GNRs in tumor tissues; Scale bar is 1 µm.

GNRs with well‐defined rod‐like structure were prepared using seed‐growth methods.^[^ ^1^, ^9^, ^15^ ^]^ The size distribution and colloidal stability (with a peak value of around 50 and 70 nm; the small peak was negligible.) were studied by transmission electron microscopy (TEM) (**Figure** **1**A) and dynamic laser light scattering (DLS) (Figure 1B). The narrow size distribution was shown in Figure 1A. GNRs were well‐dispersed in the solution with no aggregation occurred. It should be noted that the hydrodynamic size does not reflect the real size of GNRs, because the hydrodynamic size incorporates the extra space in which the neighboring particles with charged surfaces interact with each other.^[^ ^12^, ^16^ ^]^ The tumor cell specific cytotoxicity was confirmed. As shown in Figure 1C, human breast epithelial cell line (MCF‐10A, nontumor cell) and human breast adenocarcinoma (MDA‐MB‐231) were used as mode cells to evaluate the cytotoxicity and antitumor effect of GNRs. The cells were incubated with different concentrations of GNRs (0, 27, 54.0, 81.0, and 108.0 µg mL^−1^) and their viabilities were evaluated on a microplate spectra photometer using Dojindo's Cell Counting Kit‐8 (CCK‐8). GNRs displayed obvious toxicity against cancer cells (MDA‐MB‐231), while against nontumorous cells (MCF‐10A) they barely showed any harmful effects. Experiments on other cell lines, such as CHO, 293T, MCF‐7, N87 and FADU, further confirmed the specific cytotoxicity of GNRs against cancer cells (Figure S1, Supporting Information). The apoptosis and necrosis evoked by GNRs with different aspect ratios and at different time were also investigated using flow cytometer (FCM) (Figure S2, Supporting Information). GNR660 and GNR820, two GNRs with differing surface CTAB ligand densities, were selected for further investigation.

As reported,^[^ ^20^, ^21^, ^22^, ^23^, ^24^ ^]^ GNRs demonstrate good photo‐thermal conversion efficiency under the laser at 780 nm, which is highly dependent on the concentration of GNRs (Figure 1D). The in vivo accumulation and cytotoxicity were evaluated. The specific intratumor enrichment of GNRs was demonstrated by infrared thermal imaging (Figure 1E). The specific in vivo tumor inhibition effect of GNRs was studied by measuring the relative tumor volume 26 days post‐administration. The relative tumor volume of GNRs and GNRs‐NIR groups were much smaller than those in control and simple NIR groups (Figure 1F). Taken together, these data demonstrate that GNRs display specific toxicity against cancer cells both in vitro and in vivo. However, the detailed mechanisms why GNRs display specific toxicity against cancer cells remains elusive. In the study, apoptosis and necrosis were evoked through both highly GNRs accumulation in tumor cells (Figure 1G) and subsequent intracellular interaction between GNRs and organelles (Figure 1H) when GNRs were used to treat cancer cells. However, necrosis is undesirable clinically. Thus, it is necessary to examine the detailed mechanism how GNRs display specific toxicity against cancer cells, and if necrosis and apoptosis could be leveraged to benefit GNRs more in their specificity in killing cancer cells.

### 2.2 Analysis of Specific Cytotoxicity of GNRs on Extra‐/Intracellular Level

**Figure 2:**
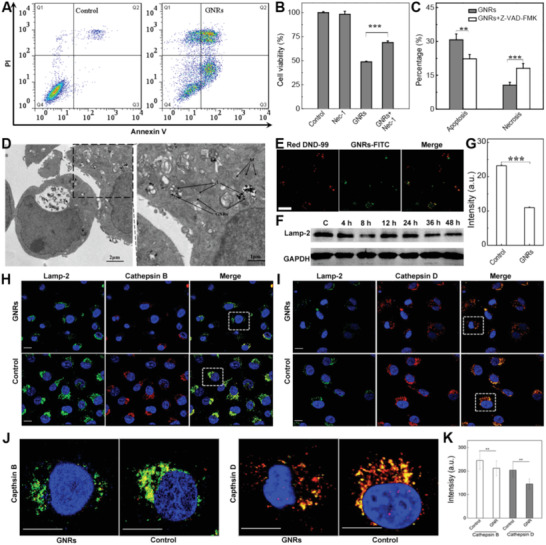
The interactions between GNRs and the organelles and the subsequent effects on the death pathways of tumor cells. A) The screen of possible death pathways induced by GNRs by flow cytometry (FCM). B) The cause of necrosis by GNRs that is evaluated by the combination of a necrosis inhibitor, Necrostatin‐1 and GNRs. C) The apoptosis induced by GNRs that is verified by the combination of a Caspase inhibitor, Z‐VAD‐FMK and GNRs as determined by Annexin V‐FITC/PI Apoptosis Detection Kit. The data represent mean value ± s.d. (*n* = 3). D) The subcellular localization of GNRs in the organelles as observed by TEM. L, M, and GNRs indicate lysosome, mitochondria, and gold nanorods, respectively. E) The localization of GNRs in lysosomes of MDA‐MB‐231 cells evaluated by CLSM. F) Lysosomal membrane permeability evaluated by the LAMP‐2 protein content checked by WB. Noted here, the GAPDH in Figures 2F and 3B,E was the same as conducted in one experiment. G) Lysosomal membrane damage checked by FCM. H,I) The changes in the lysosomal permeability determined by lysosomal membrane proteins LAMP‐2 and the dispersity of lysosomal enzyme (Cathepsin B and Cathepsin D) as imaged by CLMS. The scale bar represents 20 µm. Noted here, the GAPDH in Figures 3B,E and 2F was same. J) The enlarged picture of subcellular organelles (lysosomes) and intracellular enzymes distribution corresponding to the cells in white frame in (H) and (I). The scale bar represents 20 µm. K) The intensity of the florescence reflected the Cathepsin B and Cathepsin D as conversed from (J). Statistical significance is evaluated in panels (B), (C), (G), and (K) using an unpaired student's *t*‐test (^＊＊＊^ *p* < 0.001, ^＊＊^ *p* < 0.01, ^＊^ *p* < 0.05).

In analysis of data by convenient flow cytometry (FCM), the Q3 zone (Annexin V^+^/PI^−^) and Q2 zone (Annexin V^+^/PI^+^) are generally identical signals for early and late apoptosis, respectively.^[^ ^9^ ^]^ Moving of side population from Q2 to Q1 zone (Annexin V^−^/PI^+^) normally indicates the necrosis cells.^[^ ^15^ ^]^ The side population in Q1, Q2 and Q3 zone indicates that both apoptosis and necrosis were induced (**Figure** **2**A), which is consistent with previous reports.^[^ ^15^, ^16^ ^]^ Necrostatin‐1 (Nec‐1) and pan‐caspase inhibitors (Z‐VAD‐FMK) were used to confirm the activation of necrosis and apoptosis pathway. Nec‐1 recovered the viability of MDA‐MB‐231 cells, suggesting that GNRs induce necrosis in cancer cells (Figure 2B); While Z‐VAD‐FMK significantly reduced cell apoptosis, demonstrating GNRs’ ability to induce cancer cell apoptosis (Figure 2C). However, necrosis is expected to be as low as possible to avoid the ramification it has on cancer development, such as the recruitment of immune cells by necrosis and the subsequent promotion cancer cell growth and invasiveness.^[^ ^18^, ^25^ ^]^ Thus the key is to understand the mechanism how necrosis and apoptosis are regulated, and how without damaging GNRs’ cancer‐cell‐killing effect, necrosis could be subdued and apoptosis aggrandized.

The activation of necrosis and apoptosis by GNRs is closely related to where in the cells GNRs are located and how they interact with cellular organelles.^[^ ^19^, ^26^ ^]^ The subcellular localization of GNRs in MDA‐MB‐231 was determined 12 h post GNRs exposure. The majority of GNRs accumulated in the lysosomes (Figure 2D), where the lysosomal structures were ruptured remarkably companying with GNRs distributed in the cytoplasm. Compared with the intact nuclei of MCF‐10A, the condensed nuclei of MDA‐MB‐231 at 72 h further demonstrate the specific cancer cell killing effect of GNRs (Figure 4D). To ascertain that GNRs locate within lysosomes, we labeled GNRs with FITC (green) and studied their colocalization with LysoTracker‐Red (a specific marker for live cell lysosomes) in MDA‐MB‐231 cells. The orange color, overlapping color of red and green, confirmed the coexistence of GNRs and LysoTracker‐Red, indicating the co‐localization of lysosomes and GNRs (Figure 2D,E).

The interaction between GNRs and subcellular organelles is tightly regulated by the morphology and surface properties of GNRs, for example, the formation of protein corona post blood exposure shields the surface charge of GNRs and hinders their potential interaction with subcellular organelles (Figure S1A, Supporting Information). The low potential (0–5 Mv) of the GNRs‐FBS (FBS coated GNRs with an *R*_h_ of around 88.4 nm, Figure 1B) enhances the cellular uptake of GNRs via clathrin‐independent or caveolin‐mediated endocytosis.^[^ ^1^, ^16^, ^24^ ^]^ Following endocytosis, GNRs were engulfed by endosomes and subsequently translocate to the early lysosomes.^[^ ^1^, ^11^, ^24^, ^27^ ^]^ Structurally, the exchange between protein corona of GNRs and proteins inside lysosome may be mainly attributed to the deteriorated stability of lysosomes in cancer cells than in noncancer cells, which might result in different intracellular locations of GNRs.^[^ ^9^, ^19^, ^26^ ^]^ Lysosome, the major intracellular digestive organelle, comprises around 40 different hydrolytic enzymes, such as proteases, nucleases, and glycosidases.^[^ ^28^, ^29^ ^]^ Upon GNRs entering lysosomes, the protein corona was easily degraded, thus the re‐exposure of the cationic surface to the lysosomal membrane. The re‐exposed CTAB disrupted the integrity of lysosomal membrane, resulting in an increase in lysosomal membrane permeabilization (LMP) of GNRs.^[^ ^1^, ^11^, ^15^, ^16^ ^]^

The LMP of MDA‐MB‐231 cells were then analyzed using western blot (WB) and flow cytometry (Figure 2F,G). The obvious decrease in the intensity of fluorescent labeled LAMP‐2 indicates that the membrane was disrupted as treated by GNRs (Figure 2G). LAMP‐2 is a lysosomal membrane marker that can be used to monitor the integrity of lysosomal membrane. Expression of LAMP‐2 molecule in MDA‐MB‐231 cells further confirms the rupture of the lysosomal membrane (Figure 2F upper panel). The content of LAMP‐2 decreased as the treating time increased from 6 to 12 h. The confocal laser scanning microscope (CLSM) images also indicates that over time the number of lysosomes in normal cells increased, whereas the number of lysosomes in cancer cells decreased (Figure S3, Supporting Information).Taken together, these data demonstrate that the lysosome of MDA‐MB‐231 cells ruptured and the permeability of the lysosome membrane extensively augmented.

The lysosomes ruptured extensively in MDA‐MB‐231 cells as a result of Cathepsin B diffusion in cytoplasm (Figure 2H), which implies the activation of necrosis. Likewise, Cathepsin D also diffused significantly inside tumor cells following GNRs treatment (Figure 2I). While the increase of LAMP‐2 (green color) over time indicates an intact lysosomal membrane for the untreated cells as control, the Cathepsin B (red color) dot could also be observed.^[^ ^29^ ^]^ To evaluate the diffusion of lysosomal enzymes D and B, the enlarged CLSM image of single cell was shown in Figure 2J. In addition, the fluorescent intensity as converted from the CLSM by J. Statistical also clearly confirmed the difference (Figure 2K). Thereby, the reduction of LAMP‐2 reveals that LAMP expression is an initiating event considering Cathepsins release from the lysosomes, and plays a critical role in the activation of downstream apoptosis/necrosis pathway. The series of intracellular reactions induced by GNRs were investigated using MDA‐MB‐231, given that GNRs displayed specific cytotoxicity against cancer cells. In summary, the destruction of lysosomal membrane in tumor cells mainly accounted for the subsequent necrosis and apoptosis activity. Thus, the relationship between intracellular protein's level and properties of GNRs might be utilized to regulate cell apoptosis or necrosis due to the release of catabolic hydrolases into the cytosol.^[^ ^30^, ^31^, ^32^, ^33^ ^]^

### 2.3 The Special Intracellular Molecules Related to the Necrosis and Apoptosis

**Figure 3:**
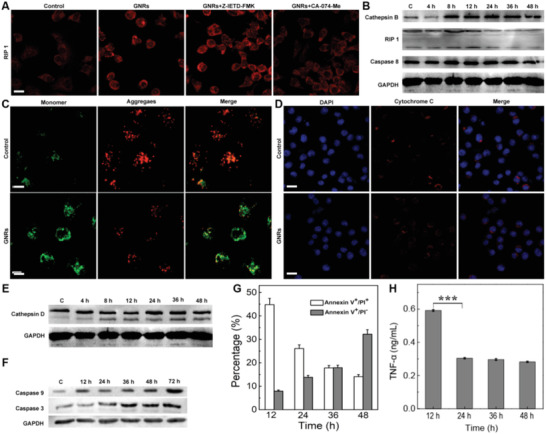
Necrosis and apoptosis caused by GNRs. A) The confirmation of necrosis and apoptosis according to immunofluorescence (IF) imaging. The apoptosis is evaluated by Caspase 8 inhibitor Z‐IETD‐FMK and Cathepsin B inhibitor CA‐074‐Me by IF. B) The molecular expression of key protein including the cleaved Caspase 8, RIPI, and Cathepsin B as tested by WB. The detailed apoptosis pathway of MDA‐MB‐231 cells detected by C) mitochondrial membrane potential according to JC‐1 assay and D) the leakage of cytochrome c induced by GNRs. E) The expression and activation of Cathepsin D released from lysosomes in tumor cells tested by WB. F) Confirmation of apoptosis based on the protein level of cleaved caspase 9, and cleaved caspase 3 tested by WB. G) The time dependence of apoptosis and necrosis checked by the Annexin V‐FITC/PI Apoptosis Detection Kit by flow cytometry for same GNRs and tumor cells. The data represent mean ± s.d. (*n* = 3). H) TNF‐*α* level as determined by ELISA. The data represent mean ± s.d. (*n* = 3). Statistical significance is evaluated using an unpaired student's *t*‐test (^＊＊＊^ *p* < 0.001). Noted here, the GAPDH in Figure 3B,E and Figure 2F was same as conducted in one experiment; the scale bar in A, C, and D is 20 µm.

The receptor‐interacting protein (RIP) is one of the serine‐threonine kinase family members that plays an important role in cell death. Aggregation of RIP1 and RIP3 is key to necrosis.^[^ ^34^ ^]^ Necrostatin‐1, a RIP1 inhibitor that inhibits the interaction between RIP1 and RIP3, could prohibit the occurrence of necrosis.^[^ ^35^ ^]^ Tumor cell necrosis was assessed by RIP1 immunofluorescence (IF) post CA‐074‐Me (a Cathepsin B inhibitor)^[^ ^36^ ^]^ treatment (**Figure** **3**A). The fluorescence of RIP1 in GNRs+CA‐074‐Me group decreased remarkably, suggesting that lysosome is the venue where GNRs starts to affect their intracellular cytotoxicity and Cathepsin B released from lysosome induces cancer cell necrosis. Expression of caspase 8 generally suppresses the formation of the necrotic complex.^[^ ^37^, ^38^ ^]^ Administration of caspase 8 inhibitor, Z‐IETD‐FMK, increases RIP1 expression (Figure 3A), reassuring that the early cell death induced by GNRs is necrosis.^[^ ^1^, ^11^, ^19^ ^]^ Variations of RIP1 and caspase 8 molecules related to necrosis were also examined by western blot (Figure 3B). The RIP1 level increased step by step reaching its maximum 12 h post GNRs treatment. Put together, these data suggest that RIP1 is an ideal protein to monitor tumor cell necrosis, and caspase 8 might be used to suppress the necrosis.

Besides Cathepsin B, Cathepsin D was released from lysosomes as well (Figure 2I). Cathepsin D facilitates mitochondrial outer membrane permeabilization (MOMP), implying that apoptosis is another death pathway that could have been induced by GNRs treatment. JC‐1 Mitochondrial Membrane Potential Assay Kit (Cayman Chemicals) was used to determine the permeability of the outer membrane of mitochondria. Aggregated JC‐1 appears to be red with high potential, indicating faulty membrane permeability; while monomeric JC‐1 appears to be green with low potential indicating good membrane permeability.^[^ ^39^, ^43^ ^]^ Here, the outer membrane of the mitochondria of MDA‐MB‐231 was stained with JC‐1 (10 µg mL^−1^) and analyzed using CLSM (Figure 3C). CLSM images display obvious green color in groups treated by GNRs, indicating high MOMP. CLSM images further showed that the mitochondrial membrane of the control group was intact (well localized cytochrome c (Cyt c) in mitochondria), while the mitochondria membrane of the cells treated by GNRs were compromised, releasing Cyt c from mitochondria to the cytoplasm. Thus, the stronger MOMP induced by GNRs could further result in higher Cyt c released into the cytoplasm (Figure 3D), which was also reflected in the expression of Cathepasin D (Figure 3E).^[^ ^39^, ^41^ ^]^ Cyt c triggers cell apoptosis via mitochondria‐dependent pathway.^[^ ^15^ ^]^ Particularly, Cyt c activates apoptosis‐related proteins, such as caspase 9 and caspase 3. To study whether Cathepsin D was involved in the apoptosis induced by GNRs, the expression levels of Cathepsin D, Caspase 9, and Caspase 3 were tested by Western blot (bottom panel in Figure 3E,F). The expression of Cathepsin D reached its maximum at 24 h, while the levels of caspase 9 and caspase 3 increased monotonically. These findings indicates that apoptosis is related to mitochondria, which is essential to lysosome‐rupture‐induced cell apoptosis.

The molecular study demonstrates that GNRs induced tumor cell death was realized mainly through necrosis and apoptosis. Apoptosis increased over time while necrosis decreased (Figure 3G). Moreover, expect for Cathepsin D and Cathepsin B, corresponding to apoptosis and necrosis separately, the lysosomes release many other enzymes and factors. Lysosomal cysteine endopeptidase, which is widely expressed, contributes to TNF‐*α*‐induced necrosis in tumor cells.^[^ ^31^, ^32^ ^]^ Figure 3H shows that the level of TNF‐*α* tested by enzyme‐linked immunosorbent assay (ELISA) decreased obviously after treated longer than 12 h, which was coincided with that of necrosis. Both RIP1 level and TNF‐*α* content confirmed that the necrosis was evoked as a result of producing the undesired inflammation factors.

It would be great to suppress the GNRs‐induced necrosis and promote apoptosis in clinical antitumor therapy.^[^ ^33^ ^]^ But there is still lack of detail intracellular pathway related to the death way. The key to overcome this issue is understanding of the detail molecular mechanism and to find the key protein related to both apoptosis/necrosis as induced by GNRs. Noted here, the inflammation factors TNF‐*α*, an important cytokine released during inflammation, was also released out, resulting in over‐expression of Caspase 8 at early stage (Figure 3B). Interestingly, over‐expression of caspase 8 promotes the expression of caspase 3 as a result of high level apoptosis.^[^ ^37^ ^]^ The activation of caspase 8 not only suppresses RIP1 formation but also activates caspase 3.^[^ ^37^, ^44^, ^45^ ^]^ Thus, the correlation between the GNR's interaction with subcellular lysosomes and the expression of caspase 8 is important to converse necrosis to apoptosis.

### 2.4 The Key Protein Level Regulated via GNRs‐lysosomes Interaction

**Figure 4:**
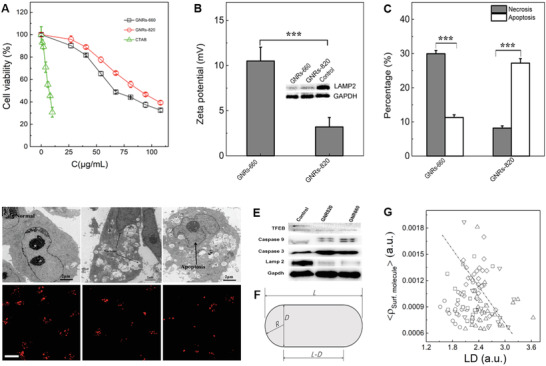
The effects of GNR's properties on the strength of necrosis and apoptosis. A) The effects of composition of surface of GNRs on the specific toxicity. B) The effects of surface properties of GNRs with distinct L/D ratio on the content of necrosis and apoptosis. Here, the corresponding LMP membrane protein level was reflected in the inserted WB. C) The relation between *L*/*D* and necrosis and apoptosis evoked by GNRs with distinct *L*/*D* ratio. The data points represent mean ± s. d. (*n* = 3). Statistical significance was evaluated using an unpaired student's *t*‐test for (B) and (C) (^＊＊＊^ *p* < 0.001). D) The detail intracellular interaction between the GNRs with distinct L/D ratio and lysosomes further confirmed by TEM and CLSM. Noted here, the scar bar in D is 20 µm. E) The revaluation of molecules caspase 9, caspase 3, and LAMP‐2 related to apoptosis, necrosis, and the key proteins of THEB for necrosis‐to‐apoptosis transition. F) The scheme illustrated the microstructural dimension of GNRs. G) The relation between *LD* and the normalized surface molecules <*ρ*_surf. molecule_> of GNRs with different aseptic ratio, the value of *LD* was analyzed from GNRs in the TEM.

**Figure 5:**
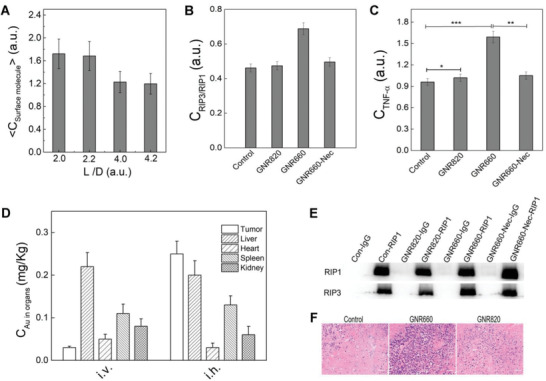
The relationship between surface molecule density and the in vitro*/*vivo cell death mechanisms. A) The aspect ratio dependence of the surface molecule concentration of different GNRs as valuated by the TGA. B) The quantitative analysis of the necrosis complex (RIP1/RIP3) formation ability induced by the different GNRs, and the inhibition of necrosis by Nec, which was converted from the WB data (as shown in the panel (E)). C) The TNF‐*α* expression as induced by the GNR660, GNR820, and GNR660‐Nec as tested by the Elisa. The analysis used an unpaired student's *t*‐test (^＊＊＊^ *p* < 0.001, ^＊＊^ *p* < 0.01, ^＊^ *p* < 0.05). D) The intratumor accumulation and biodistribution of GNRs as evaluated by ICP‐mass through the nude mice model bearing breast cancer as administrated by *i.v*. and *i.h*. The *y*‐axis was weight of Au in the organs. E) The RIP1 and RIP3 protein expression level as induced by the GNR660, GNR820, and GNR660‐Nec as tested by WB. F) The in vivo inflammatory level in tumor of PBS, GNR660, and GNR820 as evaluated by the HE staining, the blue color referred to the inflammatory level. Noted here, the scale bar was 100 µm.

The aforementioned studies have confirmed that the‐expression of caspase 8 plays an important role in the pathway of cell death. To suppress necrosis, we first seek to understand the mechanism how GNRs interact with the lysosomes. The interaction between GNRs and the lysosomes is strongly affected by the physicochemical properties of GNRs, such as their surface potential and aspect ratio. The cytotoxicity of GNR660, GNR820 as well as CTAB against MDA‐MB‐231 were re‐tested as shown in **Figure** **4**A. The half maximal inhibitory concentration (IC_50_) of GNR660, GNR820 and CTAB was 73.57, 89.08, and 6.79 µg mL^−1^, respectively. While GNR820 and GNR660 have mild cytotoxicity, CTAB was highly toxic. GNR820 displayed slightly lower cytotoxicity than GNR660 did, indicating that the content of CTAB on GNR 820 was less than that on GNR660. Actually, the loss of total weight of CTAB on GNR820 was also less than that of GNR660 was indirectly estimated in thermo gravimetric analysis (TGA) experiments (following **Figure** **5**A). Moreover, Figure 4B showed the surface potential of GNR660 is higher than that of GNR820 even after purification by centrifugation, which mainly contributes its higher cytotoxicity (Figure 4A). The higher content of CTAB resulted in stronger LMP as reflected by the lower level of membrane proteins (the insert in Figure 4B). Therefore, GNR660 mainly induces necrosis, while GNR820 mostly causes apoptosis; and the difference should ascribe to their differing surface properties, particularly the different amount of surface CTAB (Figure 4C). In addition, when CTAB was replaced by PEG, little apoptosis or necrosis was induced under the same conditions (Figure S4, Supporting Information). Note here that no NIR‐light was used in the experiments.

The interaction between the lysosomes and GNRs was further evaluated by TEM (Figure 4D). It is clear that, GNR660 group (middle) displayed more lysosome membrane rupture than GNR820 group (right) and the control group (left). This was also coincident to the high surface potential of GNR660 caused by the densely arrayed cationic CTAB on the surface. The higher the surface potential of GNRs, leads to the heavier disruption of the lysosomal membrane, and much more released proteins (bottom panel in Figure 4C). However, as shown in Figure 3B, the stronger lysosomal membrane disruption treated with GNR660 with small aspect ratio (length to diameter ratio, *L*/*D*) induced higher over‐expression of caspase 8 protases, which conversely suppressed necrosis complex R1P1 and increased the apoptosis via caspase 3 expression. Thus, the effect of GNRs on expressions of proteins related cancer cell death pathway, including caspase 3, caspase 8, and caspase 9, have been studied by WB. Additionally, the TFEB protein expression was also evaluated, because it reflected the interaction between GNRs and the lysosomes. The changes of these proteins were shown in Figure 4E, and it is clear that the higher density of the surface molecules can induce stronger the damage of the lysosomal membrane following by higher TFEB protein level. Figure 4F shows the microstructure of GNRs. Their morphology was simplified as column with or without corona covering each end.^[^ ^1^, ^7^, ^9^, ^14^, ^15^, ^16^, ^48^, ^49^, ^50^ ^]^

### 2.5 The Necrosis to Apoptosis Regulation via GNR's Surface Molecule Density

**Figure 6:**
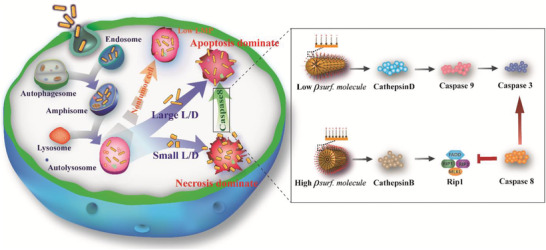
The schematic illustration about the relationship between surface molecule density and the cell death pathway. Surface density of CTAB on GNRs largely influences the internalization of GNRs, its intracellular route, the interaction between GNRs and lysosomes, and the specific killing of cancer cells dominated by apoptosis or necrosis. Caspase 8 protease plays a key role in the cell fate transition from necrosis to apoptosis which is tuned by tailoring GNR's Surface molecule density *ρ*_surf. molecule_.

The molecular mechanism how GNRs regulate the transition between necrosis and apoptosis in cancer cells was shown in **Figure** **6**. Following endocytosis, GNRs enters the lysosome, resulting in an increase in LMP. Such destabilized lysosomes specifically appeared in cancer cells, while the lysosomes remained wholesome in noncancerous cells. The disruption of the lysosomal membrane in cancer cells, resulted in the low expression of LAMP 2 level but the high level of Cathepsin B and D. The necrosis was evoked when RIP necrosis complex appeared. The Cathepsin D, released into the cytosol, translocated to mitochondria and increased MOMP, which further promoted the release of Cyt c, and triggered mitochondria‐dependent apoptosis via caspase 9 and caspase 3. Necrosis and apoptosis were induced spontaneously.

Microstructurally, the effects of GNR's surface properties, the surface molecules density (*ρ*_surf. molecule_) on the intracellular molecules related to necrosis and apoptosis, as well as the key proteins caspase 8 on the necrosis to apoptosis transformation was detailedly illustrated in the enlarged figure (right panel). Compared with the GNRs with large *L/D* and low surface potential, the GNRs with small *L/D* resulted in higher LMP because of their larger surface molecule density. According to the Figure 4F, the key parameter *ρ*_surf. molecule_ was first defined by us through the quotient between total surface molecule number (*N*_surf. molecules_) and the surface area (*S*_GNR_)

(1) $$\rho_{\mathrm{surf}.\;\mathrm{molecule}}=\frac{N_{\mathrm{surf}.\;\mathrm{molecules}}}{S_{\mathrm{GNR}}}\;\;$$

where *S*_GNR_ relates to the sphere's diameter *r* and the column's diameter *D* and height *L* − *D*

(2) $$\begin{array}{c} S_{\mathrm{GNR}}\;=\;2\;\times \;S_{\mathrm{corona}}\;+\;S_{\mathrm{column}}\;=\;2\times \frac{1}{2}\times 4\pi \gamma^{2}\;+\;2\pi \gamma \left(L\;-\;D\right)\;=\;\pi DL \end{array}$$

Thus, the final quantitative relationship between *ρ*_surf. molecule_ and the length and diameter (*L* and *D*) of GNRs was easily deduced as

(3) $$\;\;\rho_{\mathrm{surf}.\;\mathrm{molecule}}=\frac{N_{\mathrm{surf}.\;\mathrm{molecules}}}{a\pi DL}$$

Note here that the parameter a is a correcting factor. If the column wears a corona at each end, a will be 1, and *S*_GNR_ equals *πDL*. If the column wears nothing at the end, *a* is less than 1, and *S*_GNR_ would be calculated as *S*_GNR_ *= πDL+*0.5*πD^2^*. The regulation of the aspect ratio of GNRs (*L/D*) was realized mainly through changing the concentration of argentum nitricum, which indicated that the *N*_surf. molecules_ and the *D* in the system was similar.^[^ ^1^, ^14^, ^15^, ^16^, ^48^, ^49^ ^]^ We further confirmed that the difference of GNRs was mainly dominated by their *ρ*_surf. molecule_, which was directly inverse proportional to *DL* of the product. According to the TEM images of different GNRs (Figure S5, Supporting Information), the value of *LD* was analyzed by Nano Measurer, and produced a same inverse proportional relation between *ρ*_surf. molecule_ and *DL* (shown in Figure 4G). In addition, we found that *DL* of GNR‐660 was smaller than that of GNR‐820, which was also reflected in many other GNRs, and indicated that normalized surface molecule density <*ρ*_surf. molecule_> of GNRs with larger *L/D* was smaller than that of GNRs with smaller *L/D*. Although the numbers along y axis might fluctuated as the purification methods, which did not affect the inverse proportional relationship between normalized surface molecule density <*ρ*_surf. molecule_> and *DL* or *L/D*. Note here that a larger *L/D* results in a higher *DL*, because *D* is fixed for given GNRs. Additionally, such relationship would be more accurate by adding more samples with fine chemical synthesis.

The GNRs with small L/D and high *ρ*_surf. molecule_ increases cellular stress and induces over‐expression of caspase 8, which suppresses the activation of the necrosis factor RIP1 by cleaving RIP1‐RIP3 complex. Moreover, caspase 8 promotes the activation of apoptosis‐related factor caspase 3. Subsequently, both the necrosis complex RIP1‐RIP3 cleaving and the caspase 3 evoking by over‐expressed caspase 8 from GNR‐660 with high surface potential, that is, the higher surface molecules density*ρ*_surf. molecule_, dominates the necrosis‐to‐apoptosis transition. Consequently, the intracellular interaction between GNRs with lysosomes, which is strongly affected by the surface cationic molecules CTAB, plays an important role in cancer cell death. Further, the disruption of lysosome membrane due to the overexpressed caspase 8 is key to regulate undesired necrosis for application.

### 2.6 The Relation between Surface Molecule Density and the In Vitro*/*Vivo Cell Death Mechanisms

The relationship between surface molecule density and the in vitro*/*vivo cell death way including apoptosis and necrosis was further evaluated by the in vitro*/*vivo experiments. The amount of CTAB on the GNRs with different *L/D* was evaluated by TGA as shown in Figure 5A. The surface CTAB density of GNR620 and GNR660 (2.0 and 2.2) was higher than that of GNR780 and GNR820 (4.0 and 4.2), indicating that the higher aspect ratio of GNRs reduced to the lower *ρ*_surf. molecule_. Considering that both apoptosis and necrosis take place in a way that the lysosome membrane brake down and release lysosomal enzymes, including Cathepsin B and Cathepsin D. Cathepsin B mainly contributes to necrosis. The quantitatively analysis of the necrosis complex (RIP1/RIP3) formation induced by the different GNRs was re‐visited by Co‐IP (Figure 5E). The necrosis related RIP3/RIP1 converted from the WB and the TNF‐*α* expression was induced by the GNR820, GNR660 and GNR660‐Nec tested by ELISA was shown in Figure 5B,C. C_RIP3/RIP1_ decreased as the necrosis inhibitor (NEC‐1) confirmed the necrosis suppression with lower C_TNF‐_ *α*. The higher TNF‐*α* expression in GNR660 treated group was due to the higher surface CTAB density. All these results confirm that in tumor cells GNRs induced both apoptosis and necrosis. The in vivo bio‐distribution of GNRs, administrated through *i.v*. and hypodermic injection *(i.h.)* was evaluated by ICP‐mass (Figure 5D). GNRs administrated through *i.h*. showed higher intratumor accumulation than those administrated through *i.v*. Form Figure 5D,E, it is clear that GNRs did accumulate in tumor tissues. It was reasonable that tumor tissue accumulation of GNRs was lower via tail vein injection than that by *i.h*. In addition, the HE staining of tissues from groups of control, GNR660 and GNR820 shown in Figure 5F, showed that the inflammation (blue color) evoked by GNR660 was much higher than that evoked by GNR820, which further corroborates the conclusion.

## 3 Conclusion

This study determines the biological effects of GNRs in cancer cells and the molecular mechanism involved. Following endocytosis, GNRs enters the lysosome, resulting in an increase in LMP. Such destabilization of lysosomes specifically occurs in cancer cells, followed by the release of lysosomal enzymes, including Cathepsin B and Cathepsin D. Cathepsin B mainly contributes to TNF‐*α*‐induced necrosis, while Cathepsin D evokes apoptosis. The necrosis‐to‐apoptosis transition is dominated by the key protein caspase 8, which cleaves the RIP1‐RIP3 complex, activates caspase 3 and induces caspase‐mediated apoptosis consecutively. In addition, the expression of caspase 8 is strongly affected by the interaction between GNRs and lysosomes, which is determined by the surface properties of GNRs. High density of CTAB induces high cellular stress with caspase 8 over‐expression, which sets off the clinically favorable necrosis‐to‐apoptosis transition and reduces inflammation. Therefore, the intracellular interaction between GNRs and lysosome and the subsequent necrosis‐to‐apoptosis transition were mainly mediated by GNRs’ surface properties, particularly the density of surface cationic molecule *ρ*_surf. molecule_, which upon appropriate adjustment will benefit a great deal GNRs’ potential application in clinics.

## 4 Experimental Section

### Materials and Methods

The following chemicals were purchased from Sigma Aldrich: silver nitrate (AgNO_3_), sodium borohydride (NaBH_4_), hydrogen tetrachloroaurat(III) (HAuCl_4_), L‐ascorbic acid, cetyltrimethy‐ lammonium bromide (CTAB), and Fluorescein isothiocyanate (FITC); Fetal calf serum (FCS) was purchased from Gibco; CCK‐8 Kit was purchased from Dojindo Laboratories; Annexin V‐FITC/PI Apoptosis Detection Kit was purchased from Becton, Dickinson and Company; LysoTracker‐Red was purchased from Invitrogen; TNF‐*α* ELISA kit was purchased from R&D SYSTEMS; fluorochrome‐conjugated secondary antibody (Invitrogen); JC‐1 Mitochondrial Membrane Potential Assay Kit was purchased from Cayman Chemicals; The following antibodies were purchased from Cell Signaling Technology: anticathepsin B, anticathepsin D, anticaspase 8, anticaspase 9, anticaspase 3, anti‐RIP1, anticytochrome c, anti‐LAMP‐2; The following inhibitors were purchased from Selleck Chemicals: Necrosis inhibitor necrostatin‐1, caspase inhibitor Z‐VAD‐FMK, cathepsin B inhibitor CA‐074‐Me; caspase 8 inhibitor Z‐IETD‐FMK; 18.2 MΩ cm Ultrapure water produced by a Millipore Milli‐Q Plus Water Purification System from Millipore was used in all experiments. All chemicals were used as received without further purification.

### Preparation and Characterization of Nanomaterial

GNRs with different aspect ratio were synthesized by the seed‐mediated growth method.^44^ GNRs with different aspect ratio were obtained by controlling the amount of AgNO_3_. The UV–vis spectrophotometer cary300 was used to detected GNRs’ light absorption curve in GNRs‐CTAB, Purified‐GNRs and GNRs‐protein corona. The dynamic light scattering (DLS) was used to characterize GNRs’ particle size. The Malvin Zeta potential instrument (ZetasizerNano ZSP) was used to detect the Zeta potential size of GNRs‐CTAB and purified GNRs. The GNRs’ morphology characteristics were detected by TEM. GNRs were centrifuged at 10 000 rpm for 10 min and the precipitate was dispersed with equal volume ultrapure water. GNRs were centrifuged (8000 rpm, 10 min) and get the purified GNRs. Purified GNRs were incubated in the DMEM (10% FCS) medium overnight to be coated with FCS for the further evaluation of cytotoxicity.

### Cell Culture

MCF‐10A, Human breast adenocarcinoma cell line (MDA‐MB‐231), A549, and 293T were used for all experiments. Cells were cultured in Dulbecco's modified Eagle's medium (DMEM) supplemented with 10% (v/v) fetal calf serum (FCS), and cultured in a humidified 5% CO_2_ incubator at 37 °C. Cells were pre‐cultured overnight until confluence was reached to 80% before all the experiments.

### Cytotoxicity Assay

Cells were seeded into 96‐well microplates (2.0 × 10^5^ cells mL^−1^, 100 µL per well)^[^ ^46^ ^]^ and cultured for 12 h. The cells were incubated with GNRs at concentrations 27, 40.5, 54, 67.5, 81, 94.5, 108, 121.5 and 135 µg mL^−1^. Cytotoxity of GNRs was measured by using CCK‐8 Kit (DOJINDO) dispersed in DMEM with 10% (v/v). The absorbance was measured at 450 nm using a microplate spectrophotometer.

### Apoptosis and Necrosis Analysis

Cell necrosis was confirmed with Necrostatin‐1 (490 × 10^−9^ m) by CCK‐8 assay. Then the apoptosis and necrosis were further confirmed with 490 × 10^−9^ m necrostatin‐1 and 50 × 10^−6^ m Z‐VAD‐FMK by FCM. MDA‐MB‐231 cells were treated for 12 h. Then cells were washed and harvested. Cells were then tested by FCM using Annexin V‐FITC/PI Apoptosis Detection Kit (BD). The effects of influence factors including GNRs concentrations, aspect ratio and treatment time were also explored by FCM.

### GNRs and Lysosome Colocation

The CTAB‐capped Au NRs were washed at least three times, and the residual free CTAB molecules were removed from dispersion solution as much as possible. Purified GNRs were suspended in PH9.0 cross‐linking reaction solution and 75 µL FITC dissolved in DMSO (2 mg mL^−1^) was added, protected from light, incubated for 8 h, dialyzed with 3500 Da dialysis bag in pure water. MCF‐10A and MDA‐MB‐231 cells were cultured in a confocal dish. GNRs‐FITC was added and incubated for 12 h, and LysoTracker‐Red (60 × 10^−9^ m) was used to dye lysosomes by an incubation of 60 min. Then cells were imaged by CLSM with excitation wavelengths of 488 nm and 577 nm.

### Transmission Electron Microscopy Analysis

MCF‐10A and MDA‐MB‐231 cell were Continuous exposed to GNRs for 12, 36, and 72 h, respectively, and then cells were fixed with 4% polyformaldehyde, embed in resin, cut to ultrathin sections, stained with osmic acids and finally imaged on TEM.

### Lysosome Analysis

MCF‐10A, MDA‐MB‐231 cells were cultured in a confocal dish, treated with GNRs for 4, 8, 12, 24, 36, 48, and 72 h). LysoTracker Red (60 × 10^−9^ m) working fluid was added and incubated at 37 °C for 60 min, followed by imaging analysis by laser confocal microscope. At the same time, MCF‐10A and MDA‐MB‐231 cells were cultured in 24‐well plates and treated with GNRs for 4, 8, 12, 24, 36, 48, and 72 h. Lyso‐Tracker Red (60 × 10^−9^ m) working fluid was used to dye lysosomes for 60 min, The cells were harvested and analyzed by FCM.^33^

### Immunofluorescence Analysis

Cells were cultured in confocal dishes overnight and treated with GNRs for 12, 24, 36, 48, and 72 h. Then cells were fixed for 10 min on ice with methanol‐acetone (7:3) mixed liquid. The samples were then blocked in blocking buffer (1 × PBS / 5% normal serum) for 60 min and incubated with primary antibodies overnight at 4 °C. Then the samples were incubated with fluorochrome‐conjugated secondary antibody (Invitrogen) for 1–2 h at room temperature in dark, And imaged by CLSM with excitation wavelengths 488 and 555 nm.^[^ ^47^ ^]^

### Western Blot Analysis

Cells were seeded in 6‐well plates and treated with GNRs for 12, 24, 36, 48, and 72 h. Then cells were lysed by adding 1 × SDS sample buffer, harvested, heated at 95–100 °C for 5 min, cooled on ice, stored at −20 °C. Protein samples were separated by 10% SDS (sodium dodecyl sulfate)‐polyacrylamide gel electrophoresis (SDS‐PAGE) and electroblotted onto polyvinylidene difluoride (PVDF) membranes. Then the membranes were blocked, incubated with primary antibodies at 4 °C overnight, followed by incubation with secondary antibodies for 1–2 h. Subsequent chemiluminescence (Merck Millipore) detection was performed using the ECL detection system and a molecular imager (Chemi‐Doc XRS system, Bio‐Rad). The necrosis by Co‐IP for testing the necrosis related RIP1, RIP3 complex. It should be noted here that the WB data in Figures 2B, 3B,E,F, and 5E were conducted at the same time. So the reference of these figures used the same GAPDH.

### Mitochondrial Membrane Potential Analysis

Cells were cultured in confocal dishes and treated with GNRs for 24, 48, and 72 h. Then the cells were stained with 10 µg mL^−1^ JC‐1 at 37 °C for 20 min and analyzed by CLSM. The JC‐1 monomer was detected at FITC emission channel (522–535 nm) and JC‐1 aggregate was measured at RFP emission channel (560–615 nm) with the same laser excitation of 488 nm.

### TNF‐*α* Elisa Analysis

Macrophages were obtained by intraperitoneal injection in C57 mice. MDA‐MB‐231 cells were treated with GNRs for 12, 24, 36, and 48 h, followed by treatment with 2 × 10^5^ macrophages for 12 h. Then TNF‐*α* was tested using ELISA (Shanghai Senxiong Biotech Industry Cd., Ltd.).

### The In Vivo Therapeutic Efficiency

Human breast epithelial cell line (MCF‐10A, nontumor cell) and human breast adenocarcinoma (MDA‐MB‐231) were used as models to evaluate the cytotoxicity and antitumor effect of GNRs which were similar to the previous works.^[^ ^40^, ^42^ ^]^ Nude mice (4 weeks old) were purchased and divided into 4 groups. The first two groups were used as controls conducted under or not NIR laser. Another two groups were treated with GNRs, among which, one was lightened by NIR laser while another without light. The tumor cells were subcutaneously seeded in the forearm armpit region (800–1000 × 10^4^ cells per mice). The mice were treated with 200 µL PBS, 200 µL PBS with three runs NIR irradiation (1200 w, 2 min run^−1^), 200 µL GNRs at 100 µg mL^−1^, and 200 µL GNRs at 100 µg mL^−1^ with three runs NIR irradiation (1200 w, 2 min run^−1^). The thermal imager was used to analysis of the in vivo GNRs distribution. The relative tumor volume (the ratio of tumor volume at testing time to the tumor volume at 0 day). Some of the tumors were used to further intracellular distribution of GNRs. The intratumor accumulation and bio‐distribution in vivo of GNRs as evaluated by ICP‐mass with treated tissue homogenates. HE staining sections of PBS, GNR660 and GNR820 were evaluated the in vivo inflammatory level in tumor. Noted here, all of the experiments were performed in accordance with ethical guidelines under the protocols approved by the Committee of Animals of the Second Military Medical University (Shanghai, China).

### Statistical Analysis

Data were expressed as means ± SD of at least three independent experiments. Statistical analysis between negative control and treated samples was performed by one‐way ANOVA followed by Dunnett's test, and the comparison between GNRs treated and untreated cells was performed by unpaired two‐tailed Student's *t*‐test. Differences were considered significant when *p* < 0.05.

## Conflict of Interest

The authors declare no conflict of interest.

## Supporting information

Supporting Information

## Data Availability

Research data are not shared.
