# Supplementary material for: Death Pathways of Cancer Cells Modulated by Surface Molecule Density on Gold Nanorods
Source: Adv Sci (Weinh). 2021 Sep 15;8(22):2102666. doi: 10.1002/advs.202102666 (PMC8596106; doi:10.1002/advs.202102666)
Supplement: Supplementary file 1 — Supporting Information [file ADVS-8-2102666-s001.pdf]

© 2021 Wiley-VCH GmbH

ADVANCED SCIENCE
Open Access

Supporting Information

for *Adv. Sci.,* DOI: 10.1002/advs.202102666

Death Pathways of Cancer Cells Modulated by Surface Molecule Density $\rho_{surf.\ molecule}$ on Gold Nanorods

Fulei Zhang,[1,] *Yi Hou,*[1,] *Minhui Zhu,*[2,] *Bo Deng,*[3,] *Mengxin Zhao,*[1] *Xiandi Zhu,*[1] *Yun Sun,*[1] *Di Chen,*[1] *Cheng Jiang,*[1] *Liming Wang,*[5] *Chunying Chen,*[5] *Huaiwen Chen,*[2] *Han Chen,*[4,*] *Hongliang Zheng,*[2,*] *Wei Li*[1,*]

Death Pathways of Cancer Cells Modulated by Surface Molecule Density $\boldsymbol{\rho_{surf.\ molecule}}$ on Gold Nanorods

Fulei Zhang,[1,#] Yi Hou,[1,#] Minhui Zhu,[2,#] Bo Deng,[3,#] Mengxin Zhao,[1] Xiandi Zhu,[1] Yun Sun,[1] Di Chen,[1] Cheng Jiang,[1] Liming Wang,[5] Chunying Chen,[5] Huaiwen Chen,[2] Han Chen,[4,*] Hongliang Zheng,[2,*] Wei Li[1,*]

[1]*Departent of Nanomedicine & International Joint Cancer Institute, Naval Medical University, Shanghai 200433, China*
[2] *Department of Otolaryngology Head & Neck Surgery, Shanghai Changhai Hospital, the Navy Military Medical University, 168 Changhai Road, Shanghai 200433, China*
[3] *State Key Laboratory of New Textile Materials and Advanced Processing Technologies, Wuhan 430073, China*
[4] *Department of General Surgery, Navy No.905 Hospital of Chinese People's Liberation Army, Navy Military Medical University, Shanghai 200050, China*
[5]*CAS Key Laboratory for Biomedical Effects of Nanomaterials and Nanosafety, Institute of High Energy Physics and National Center for Nanoscience and Technology of China, Chinese Academy of Sciences, Beijing 100049, China*

[#]The authors contributed equally.

[*] Corresponding authors：
Prof. Chen H.: chenhan0903@163.com
Prof. Zheng H.: zheng_hl2004@163.com
Prof. Li W. : liwei_dds@163.com
Tel.: +86-21-81871644, Fax: +86-21-81870801

Keywords: Gold nanorods, Surface molecule density, Cancer therapy, Cell death pathway.

S1:

In UV testing, GNRs shows two distinct absorption peaks: a longitudinal plasmon absorption peak (inside the feux rouges region, 620 nm~710 nm) and a transverse plasmon absorption peak (at ~520 nm). This is in according with reported morphology-correlated absorption of GNRs. Proteins are reported to be easily adsorbed onto the GNR's surface by forming a protein corona. A 16 nm red shift of the longitudinal SPR peak and decreased peak intensity inside visible and near-infrared absorption spectra proves the formation of protein corona. The corresponding specific cytotoxicity mechanism, particular for detailed cell apoptosis and necrosis pathways, is still unclear considering the directly affection of the CCK-8 assay on the cell viability.

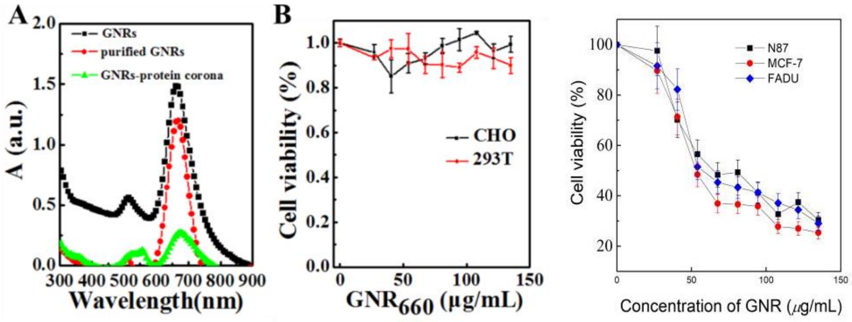

Figure S1. (A) The light absorption wavelength curve of GNRs original solution, purified GNRs solution, GNRs-protein corona solution. (B) GNRs worked on CHO, 293T Cell MCF-7, N87 and Fadu with different with different concentration.

S2

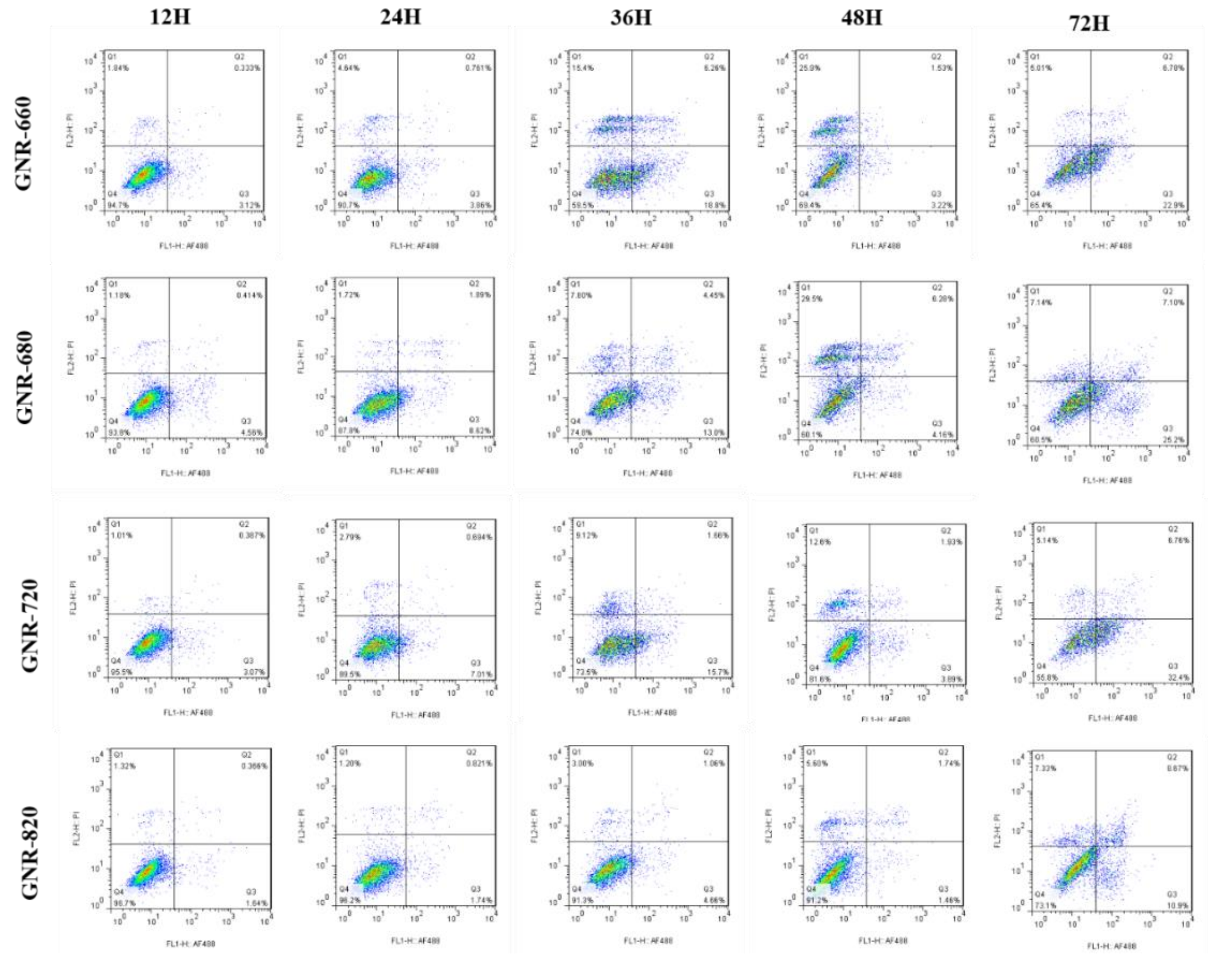

Figure S2: the apoptosis and necrosis evoking by GNRs with different aspect ratio and at different time was also investigated by flow cytometer (FCM).

S3:

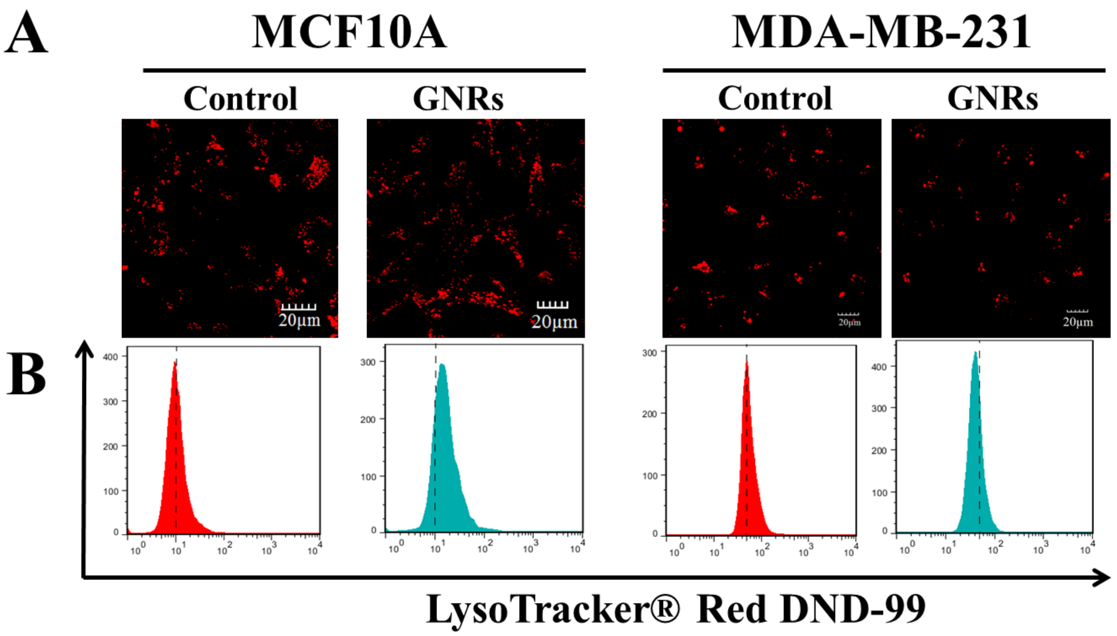

Figure S3. The lysosome quantity changed in non-cancerous cells and cancerous (A) With GNRs treating, the lysosome increased in MCF10A cells and decreased in MDA-MB-231 cells measured by CLSM.(B) With GNRs treating, the lysosome increased in MCF10A cells and decreased in MDA-MB-231 cells measured by flow cytometry..

Figure S4. CTAB without particles to see if it induces apoptosis or necrosis

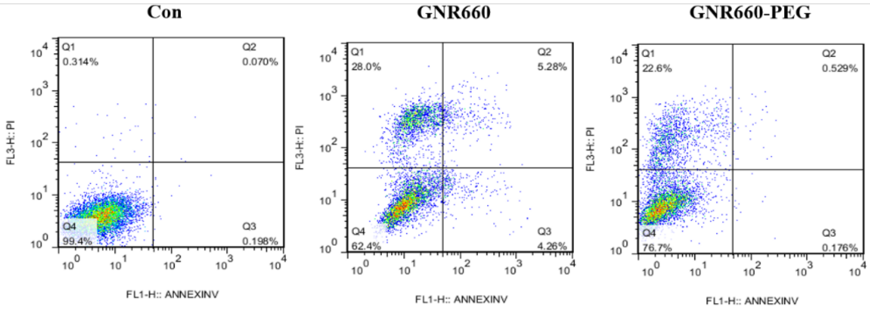

Figure S5.

TEM images of different GNRs for the L/D and LD analysis.

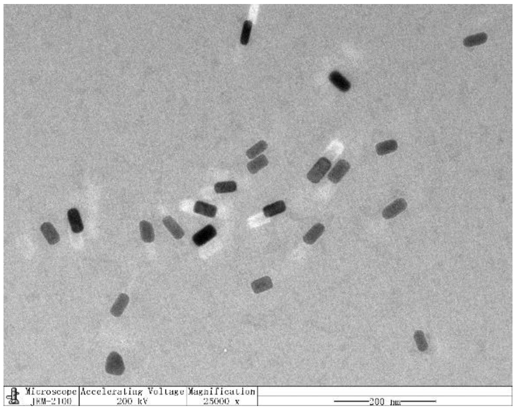

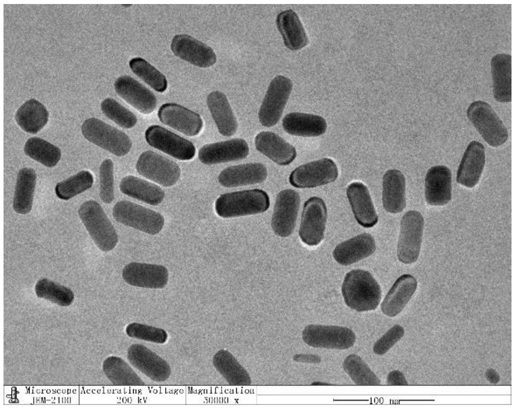

GNR620 GNR640

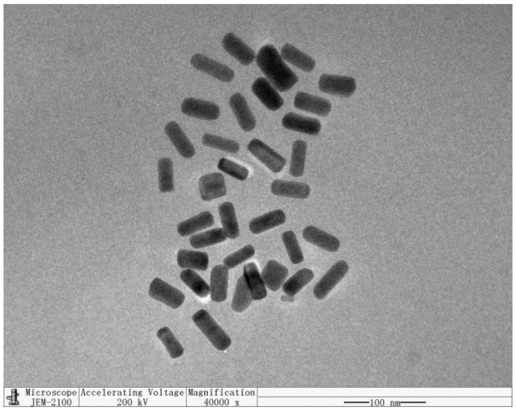

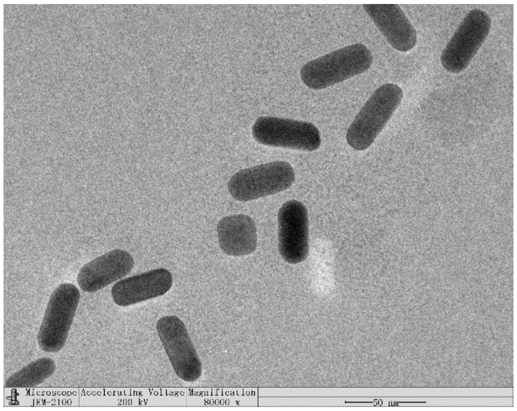

GNR660

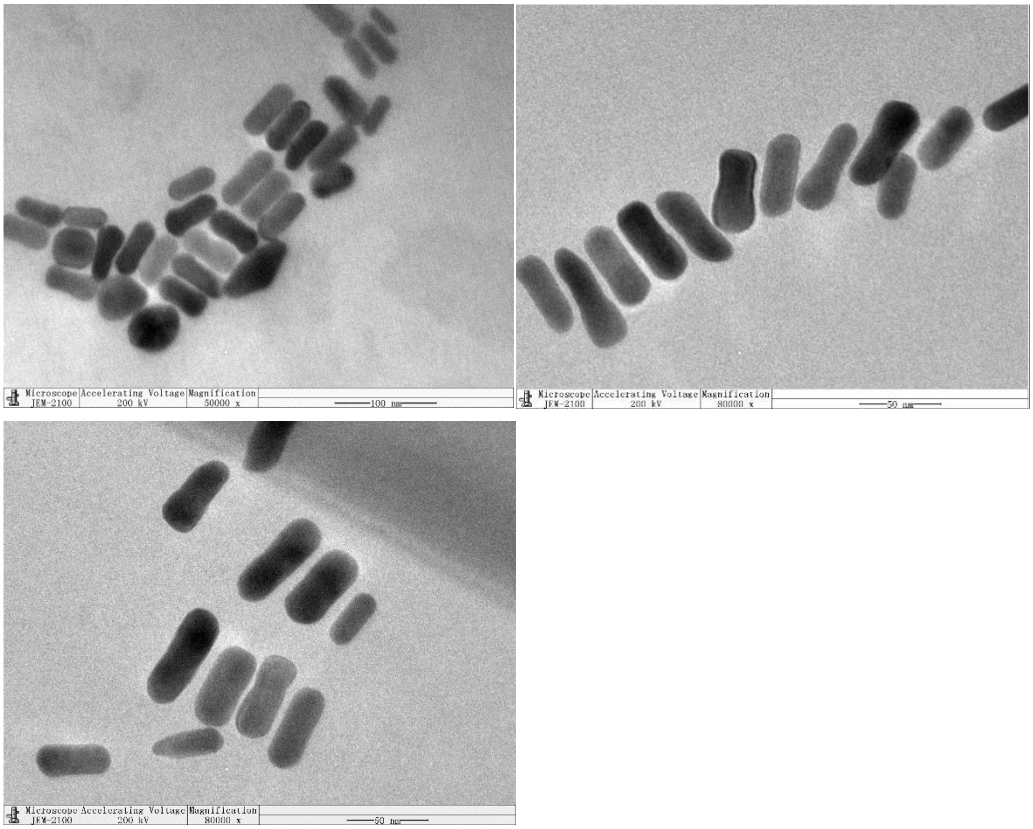

GNR680

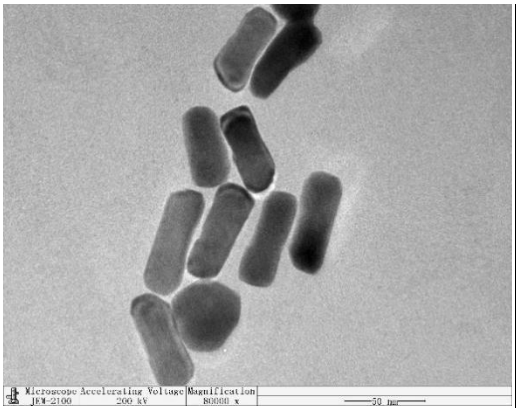

GNR710

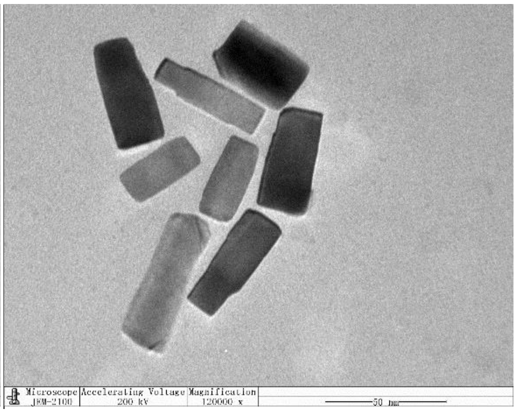

GNR710
